# On the Nature of Death from Chloroform

**Published:** 1860-10

**Authors:** Charles Kidd

**Affiliations:** 43 Sackville Street, Piccadilly.


### ARTICLE XIV.

### *On the Nature of Death from Chloroform.*

The following tables are the result of a suggestion made to me by one of our most eminent hospital surgeons, my friend Mr. Paget, who, having done me the favor recently to read my little book on Chloroform, expressed a wish that I would "compare the number of deaths after small and after great operations, to see whether the proportions of their several numbers be different from that of their several chloroform mortalities." And, Mr. Paget adds, "Your records would easily decide the question, and it is important to do so." I had said, (p. 63,) which Mr. Paget doubted, that patients bear large operations under chloroform best; that operations on sphincters, and tendinous sheaths about the fingers and toes, (p. 192,) were probably to be regarded with more dread under chloroform than the largest amputation, or such a terrible operation as ovariotomy. I may say here that Professor Murphy, also, who has seen a great deal of chloroform in general as well as in obstetric practice, was also struck by the same apparent anomaly, and wrote to me to say that it would require very rigorous examination before it could be received as a general law or fact, that deaths from chloroform are more frequent from small doses, and in trivial operations, rather than where large doses of chloroform had been taken. All I can now say is, that, however it is to be explained, there is no longer any doubt on the matter whatever, as any one may convince himself from an inspection of the following tables drawn up with some care.

There have been about 125 deaths from anæsthetics in Europe up to the present date. A very large number, the only list of any such cases on the continent, is one by M. Scoutetten, an army surgeon, who gives forty deaths, but unfortunately does not specify the nature of the operation for which the anæsthetic was administered. Dr. Snow gives fifty deaths, and I have since myself made out thirty-five deaths in addition. All agree as to the vast preponderance, however, of death in male patients rather than in females; it also appears that while there have been in reality no deaths as the result of large amputations, resections, ovariotomy, &c., fully two-thirds of the deaths have been from operations on sphincters, or tendinous sheaths, strabismus, tooth-drawing, &c.

| Nature of Operations causing death. | Snow. | Kidd. |
|---|---|---|
| 17 Deaths during operations on fistulæ, hæmorrhoids, application of caustics, &c. . . . . . . . . . | 13 | 4 |
| 14 Deaths in removal of toe-nail, dead finger, and such operations on phalanges, . . . . . . . . . . | 9 | 5 |
| 10 Dentists' cases of tooth-drawing, &c. . . . . . | 6 | 4 |
| 11 Deaths from removal of tumors, . . . . . . | 7 | 4 |
| Resections, largea mputations, midwifery, ovariotomy, ligatures of large arteries, &c., . . . . . . | — | — |
| 9 Deaths in minor amputations (but six occurring before the operation commenced,) . . . . . . . . . . | 4 | 5 |
| 6 Deaths in strabismus, operations on eye, &c., cysts of eyelids, &c., . . . . . . . . . . . | 2 | 4 |
| 9 Deaths in operations on testis, sounding, &c., (2 included doubtful in 218 lithotomy cases,) . . . . . . | 6 | 3 |
| 5 Deaths from reduction of dislocations, . . . . | 2 | 3 |
| 1 Death from strangulated hernia, . . . . . . . | 1 | — |
| 3 Deaths from delirium tremens, . . . . . . . | — | 3 |
| | 50 | 53 |

Scoutetten's cases (not specified) . . . . . . . . 40

This table explains itself; the general result is very curious, and I think very convincing, that in the hundreds of thousands of operations where chloroform has now been used, it has proved in the vast majority to be perfectly safe, and that it is safer in large than in small operations. Why it is so, I may not now stop to inquire. Again, of sixteen deaths under chloroform, where I was myself able to trace out the probable cause of the fatal issue, I found three where some remote disease of the heart might be suspected; but in thirteen there had been probable, and in some very serious derangement of the nervous system—viz. three deaths from delirium tremens and chloroform combined, two where chorea and meningitis were present, two from cysts in the brain, and four from other lesions of cerebral tissue. It seems too evident, I think, that if syncope or "fit" should occur in any such case, resuscitation is rendered difficult, as we have an imperfect brain and spinal apparatus to work with. Disease of the heart is a very rare cause of death from chloroform; the facts on which this hypothesis was built up are now shown to be all erroneous.

There is a sad want of uniformity in the mode of making post-mortem examinations in our London hospitals. In two necropsis in the borough hospital, and one at the Royal Opthalmic, Moorfields, I was present, and anxious to see the state of the heart and pericardium before the latter was opened; in two I was successful, but in the third the head was opened first, and a large drainage of fluid took place from the jugular veins, sinuses, &c., which left the heart flaccid. Even in one of the other cases, where in good routine fashion the hospital porter opened roughly all the three cavities at once, and left them all open for an hour, awaiting some "crowner's quest," or magisterial inquisition, it was not less difficult to come to any steady or useful decision on the case. I observed, however, while awaiting this hour, that the right cavities of the heart were very much distended with fluid blood; the pericardial contents and pericardium bulging very prominently, "filled to bursting almost," till the examination of the lungs commenced, when the section of the large vessels of course emptied the heart; the heart being then examined was marked "perfectly healthy—and empty." It seems to be not a bad mode of proceeding to open all the cavities together, but our great want in the hospitals is some uniformity; and in a very painful degree some uniformity in the examination of dead bodies after accidents from chloroform. Some other remarkable facts have been discovered during the course of these investigations, as to death from chloroform, which it is of interest to note. First, that a very large number of the twenty-five deaths from the use of anæsthetics after operations have been deaths from the effects of the slow administration of ether, or ether and chloroform mixed, but not from pure chloroform. I published nineteen deaths from ether two years ago, yet it is still suggested in America, that there have been no deaths from this agent. Scoutetten gives five; and since then there have been at least four or five more from ether or amylene; so that on the whole, there appears at present about a hundred deaths from pure chloroform, and one-fourth (or twenty-five) from ether or amylene! Next, as to the stage or degree of the anæsthetic process which appears most dangerous, this (contradictory though it seems) is decidedly the stage of excitement, or the early stage of violent plunging, before the patient is rendered anæsthetic and fit for the operation he is about to undergo. This escaped the knowledge of Dr. Snow.

| | Snow. | Scoutetten. | Kidd. | Total. |
|---|---|---|---|---|
| In 121 deaths the relative dangers of the chloroform "Stages" were : | | | | |
| Deaths when chloroform was given for intended operations, or immediately before operations, . . | 18 | 22 | 14 | 54 |
| Deaths during the progress of operations, . . . | 22 | 6 | 14 | 42 |
| Deaths after operations—*i. e.* from chloroform immediately after, or the result of chloroform and the operation combined a short time after the operation had been completed, . . . . . . . . | 6 | 12 | 7 | 25 |
| In 133 deaths the relative frequency of deaths as to sex was : | | | | |
| Males, . . . . . . . . . | 30 | 32 | 28 | 90 |
| Females, . . . . . . . . . . | 20 | 16 | 7 | 43 |

I will only say in conclusion, that I look on this suggestion of Mr. Paget as very valuable, and these results as most unexpected. I am, &c.,

CHARLES KIDD, M. D.

43 Sackville street, Piccadilly.

---

P. S.—I think it will be found that the mode of death by "fits," trachelismus, syncope, &c., observed before operations occurring suddenly, and consequently the means to be adopted for resuscitating patients in such accidents, differ very widely from the mode of death observed and means to be adopted for resuscitation of patients who show a tendency to sink after operations (by asphyxia?) We have hitherto confounded the two, and have been looking for some one great secret cause of these most melancholy occurrences. One set of accidents seem to occur suddenly from chloroform; the other slowly, but from ether. It appears also very instructive and consoling that in all the large tedious operations,—such as resections, large amputations, midwifery, ovariotomy, ligatures of large arteries, &c.—such is the "law of tolerance" of chloroform, that in these formidable operations it is almost free from danger; whereas, in trivial operations, (where, fortunately, we might use Dr. Arnott's congelation plan) the chances of accident are very serious indeed.

*British Jour. of Dent. Science.*

